# The Burden of Respiratory Viruses and Their Prevalence in Different Geographical Regions of India: 1970–2020

**DOI:** 10.3389/fmicb.2021.723850

**Published:** 2021-08-31

**Authors:** Rushabh Waghmode, Sushama Jadhav, Vijay Nema

**Affiliations:** Division of Molecular Biology, ICMR-National AIDS Research Institute, Pune, India

**Keywords:** respiratory viruses, India, prevalence, outbreaks, multiplex detection

## Abstract

As per the 2019 report of the National Health Portal of India, 41,996,260 cases and 3,740 deaths from respiratory infections were recorded across India in 2018. India contributes to 18% of the global population, with severe acute respiratory infection (SARI) as one of the prominent causes of mortality in children >5 years of age. Measures in terms of the diagnosis and surveillance of respiratory infections are taken up globally to discover their circulating types, detect outbreaks, and estimate the disease burden. Similarly, the purpose of this review was to determine the prevalence of respiratory infections in various regions of India through published reports. Understanding the pattern and prevalence of various viral entities responsible for infections and outbreaks can help in designing better strategies to combat the problem. The associated pathogens comprise respiratory syncytial virus (RSV), rhinovirus, influenza virus, parainfluenza virus, adenovirus, etc. Identification of these respiratory viruses was not given high priority until now, but the pandemic of severe acute respiratory syndrome coronavirus 2 (SARS-CoV-2) has sensitized our system to be alert about the burden of existing infections and to have proper checks for emerging ones. Most of the studies reported to date have worked on the influenza virus as a priority. However, the data describing the prevalence of other respiratory viruses with their seasonal pattern have significant epidemiological value. A comprehensive literature search was done to gather data from all geographical regions of India comprising all states of India from 1970 to 2020. The same has been compared with the global scenario and is being presented here.

## Introduction

The National Health Portal of India reported in 2019 that there were 41,996,260 cases and 3,740 deaths from respiratory infections in India in 2018. Acute respiratory infections (ARI) accounted for 69% of the total cases of communicable diseases, and this scenario is before the era of severe acute respiratory syndrome coronavirus 2 (SARS-CoV-2). After the coronavirus disease 2019 (COVID-19) pandemic, the total number of infected numbers rose to millions and cases are still getting added while this review was being written. The spread of the virus was so fast and wide that the World Health Organization (WHO) had to declare this infection as a global pandemic on March 11, 2020. The United States, India, Brazil, France, Russia, Spain, Argentina, the United Kingdom, Colombia, and Mexico were the countries that were impacted severely. The United States has been reporting the largest number of cases with 35,283,729 and over 626,668 deaths, followed by India with 31,341,507 and over 420,196 deaths as per data until July 24, 2021 (Worldometer, 2021).

India hosts a population of 1.3 billion people distributed in states having diverse geographical and cultural makeup. In India, the eight states with the highest total cases of SARS-CoV-2 infections are in Maharashtra, with over 3.84 million confirmed cases, followed by Karnataka, Andhra Pradesh, Tamil Nadu, Kerala, Delhi, Uttar Pradesh, and West Bengal. As expected, with the huge population of the country, these states also have a huge population. Variations in the environmental conditions, food habits, and social practices all contribute to the diversity in the distribution of respiratory infections in different ways and with different intensities (Salvi et al., 2018). The global figures indicate that acute lower respiratory infection (ALRI) is the single largest infectious cause of death among children. In India, pneumonia itself causes 17% of all deaths in children >5 years. This leads to the initiation of multiple health measures and guidelines for infants and younger children. However, being such a vast and diverse country, there remain so many gaps in the treatment, vaccination, empirical use of available antimicrobials, etc. (Krishnan et al., 2019).

Leaving aside the influence of COVID-19, the world has always been struggling with the prevalence and the diversity of respiratory infections. These are highly dependent on seasonality and the change in climatic conditions. Hence, they visit again and again in the form of epidemics in sporadic regions in the world, sometimes crossing boundaries and becoming pandemics as well. The world faces more than 2–5 million cases every year, with deaths ranging from 290,000 to 650,000 (World Health Organization, 2021). The exact cause of such a large number has not been fully understood, but 99% of cases are reported in children below 5 years and are occurring in developing countries. Hence, it has something to do with nourishment and the availability of medical facilities along with sophistication.

### Diagnostic Scenario and Prevalence of Respiratory Viruses

Diagnosing a particular respiratory viral infection based on the symptoms is difficult as all respiratory viral infections have overlapping symptoms. Testing for the presence of viruses in human samples using specific nucleic acid sequence detection is the only way to confirm diagnosis. Polymerase chain reaction (PCR) and its variants are the mainstream diagnostic modalities available, and the same is done with a single set of primers or multiplex formats after the design of specific probes and primers and optimization in the laboratory. Immunofluorescence assays are also in use, but suffer issues like sensitivity. Confirmation by culture has other issues, such as the availability of sophisticated cell culture laboratories and the containment levels available at different resource settings (Malhotra et al., 2016). Surveillance for influenza has been taken up by many countries wherein the use of multiplex real-time PCR assays has helped in detecting circulating viruses and outbreaks and estimating the disease burden. Following the development of newer methods and their optimization, more and more viruses are being diagnosed routinely or as and when suspected in many countries now. The following viruses are being detected for respiratory infections using multiplex real-time PCR methods.

### Human Respiratory Syncytial Virus

Human respiratory syncytial virus (HRSV)/RSV is considered to be the most common viral cause of ALRI-related death. It is estimated to have 33.1 million cases globally, with about 10% hospitalizations and death of 59,600 children below 5 years of age every year. This amounts to about 22% of all episodes of ALRI and is the cause of the highest childhood mortality in low- and middle-income countries among all respiratory viral infections (Broor et al., 2018). RSV infections follow a distinctive seasonal pattern, but differ according to the geographical location. The tropical or warm climate of the Southeast Asian region allows it to stay longer, with a few areas harboring the virus and its outbreaks all through the year. India is also witnessing an increase in RSV infections in infants and children below 5 years of age. Hospital-based reports indicate that RSV is detected in around 16% of children presenting acute lower respiratory tract infections (Kini et al., 2019).

### Human Parainfluenza Virus

Human parainfluenza virus (HPIV) is a virus from the family Paramyxoviriae with two genera: *Respirovirus* (HPIV-1 and HPIV-3) and *Rubulavirus* (HPIV-2 and HPIV-4). Not much could be found in the literature about the global burden estimates for the parainfluenza virus. Literature from Asia reported a variable prevalence of HPIV ranging from 1 to 66%, with 6.95% as the Indian average prevalence (Rafeek et al., 2020).

### Human Metapneumovirus

Respiratory virus infections are highly prevalent in Southeast Asian countries, but the discovery of metapneumovirus occurred in 2001 in patients from the Netherlands (van den Hoogen et al., 2001). This virus was detected from all continents and in all age groups. Reports from Hong Kong, Japan, Korea, Thailand, etc., also described detections of human metapneumovirus (hMPV) after 2001. Rao et al. (2004) have presented a preliminary report on the first detection of hMPV in India from a pediatric patient in July to August 2003 by reverse transcription PCR.

### Influenza Virus

Infection with the influenza virus is prevalent worldwide and is known to cause around 39 million episodes annually. India, with a tropical climate and variable hygiene practices, experiences a significant number of cases every year. Two strains of this virus are mainly detected: influenza A (INF-A) and influenza B (INF-B). Multiple strains of influenza have emerged in due course, and subtypes H3N2 and H1N1, belonging to INF-A, are the significant ones. Although influenza virus infections are reported throughout the year, a higher number of cases are reported during the rainy season in India. Hence, the higher relative humidity has a role to play in its incubation and spread (Narayan et al., 2020). Influenza B, which is diverse from INF-A virus, comprises two antigenically distinct lineages (“B/Victoria/2/87-like” and “B/Yamagata/16/88-like,” termed Victoria and Yamagata, respectively). This virus has also caused a few major pandemics in known history and is supposed to have appeared before the period of reporting.

### Human Rhinovirus

Rhinovirus, although belonging to the Enterovirus family, causes respiratory infections along with gastroenteritis and has been a causal factor for community outbreaks and pediatric respiratory infections in many countries across the globe. Molecular analysis of the collected strains has revealed their presence for the last 250 years all around the world (Briese et al., 2008).

### Human Adenovirus

Adenoviruses are not often reported to cause severe illnesses in normal or healthy individuals; however, they cause a wide range of illnesses in children and immunocompromised individuals. They start from respiratory symptoms such as the common cold, sore throat, bronchitis, and pneumonia to gastrointestinal disorders such as diarrhea, vomiting, nausea, and stomach pain. They are also known to cause conjunctivitis in children. Not many epidemiological reports are available from India. A report describing pediatric gastroenteritis caused by adenovirus was found in Kolkata, India. This study described the testing of 1,562 stool specimens in 2013–2014 and reported an 11.8% prevalence of enteric HAdV (Banerjee et al., 2017).

There are limited reports available regarding the global and regional burdens of all these respiratory virus infections. For instance, reports on the influenza virus and respiratory syncytial virus (RSV) are available for the global scenario, but not much could be found on the prevalence and coverage of the health system of the parainfluenza virus and metapneumovirus. Availability of reliable data is a primary requirement for the assessment of medical needs and also in the field of prediction for respiratory viral infections. Unfortunately, this is lacking in most countries, including India. We tried to assess this data gap and, hence, reviewed the literature from 1970 to 2020 mentioning cases of respiratory virus infections in India and from a global perspective. This review is all about the respiratory infections that occurred due to virus infections. It is high time that we should take some measures to manage these infections. Currently, every researcher is compelled by the circumstances to look at COVID-19 infections, although other respiratory infections should also be taken care of to avoid similar situations to other viral entities. With the COVID-19 pandemic, it has become clear that there exist deficiencies in the system and planning at a larger scale to address population-level needs. This broad evaluation of the burden of respiratory viruses in India from 1970 to 2020 points to the clear need for better and conclusive diagnostics for respiratory viruses and also for plans in controlling the spread and reemergence of infections. To our knowledge, this is the first systematic analysis of the prevalence of multiple respiratory viruses such as influenza virus, RSV, parainfluenza virus, metapneumovirus, adenovirus, rhinovirus, etc. covering all geographical regions and states of India. We also tried to cover the situation on a global scale in comparison with the Indian scenario.

## Materials and Methods

### Search Strategy

We conducted data gathering of peer-reviewed papers indexed in PubMed and available search engine reports for estimations of the burden of chronic respiratory diseases; the prevalence of respiratory viruses; and viral infection throughout the country using the keywords “prevalence,” “burden,” “respiratory viruses,” “chronic respiratory disease,” “co-infection,” “epidemiology,” “India,” “morbidity,” “mortality,” and “trends” from December 2020 to April 2021. We also tried to look for the diagnostic scenario of the virus by going through the number of publications and the types of virus reported. Our search remains restricted to the English language only. Studies with overlapping populations were sorted, and the ones with the largest and inclusive data were chosen to have conclusive outcomes.

### Eligibility Criteria

Studies with cross-sectional, prospective, and cohort designs were all included. The objective of these studies was about detailing the prevalence of respiratory viruses and respiratory viral infections. Also, studies with diagnostic intent for viral infections describing PCR, real-time PCR, and cell culture-based detection were considered. Most of the studies with pediatric respiratory infections were given priority, but infections reported in adult populations were also considered. Studies reporting on the diagnosis of multiple viral species were also given priority as they have good coverage of all respiratory viruses. Reports describing the detection of individual respiratory viruses were considered for adding the counts to the overall prevalence. Studies with animal experiments and *ex vivo* and toxicological perspectives were excluded, along with non-peer-reviewed articles and reports.

## Results

### Prevalence of Respiratory Viruses in India Based on Reported Cases

During our literature survey, we decided to gather data on reviews regarding the combined prevalence of respiratory viruses in India; however, no recent data could be found on Google Scholar, PubMed, and other search engines accessible to us. Hence, segregated data from different regions and states or cities in India were gathered and combined. A total of 35 research articles from 1970 to 2020 were considered significant, with around 22,000 cases examined (Table 1). The reports in terms of reliable publications did not emerge in a sustained fashion. Rather, there have been gaps in reporting. This was due to the unavailability of a program that could have monitored the prevalence of these viruses on a yearly basis. As apparent from Table 1, not many reliable reports could be found describing viral prevalence during 1970–2000. A similar trend was found from 2001 to 2007. However, with the availability and affordability of PCR and other detection modalities, regular reporting was observed after 2007. The two peaks in numbers, as observed in 2009 and 2012, were due to outbreaks, and hence there was enhanced diagnosis during those periods. This change in number was found to be an outcome of a change in the diagnostic rate. Earlier diagnoses or surveys did not have a comprehensive panel of viruses, and therefore the trend shows no detection of a few viral entities such as rhinovirus and hMPV in initial reports. Influenza, being an annual affair and better known to diagnostic systems, was always included in the diagnosis and was always detected in higher numbers.

**TABLE 1:** Summary of cases studied from various states/cities of India.

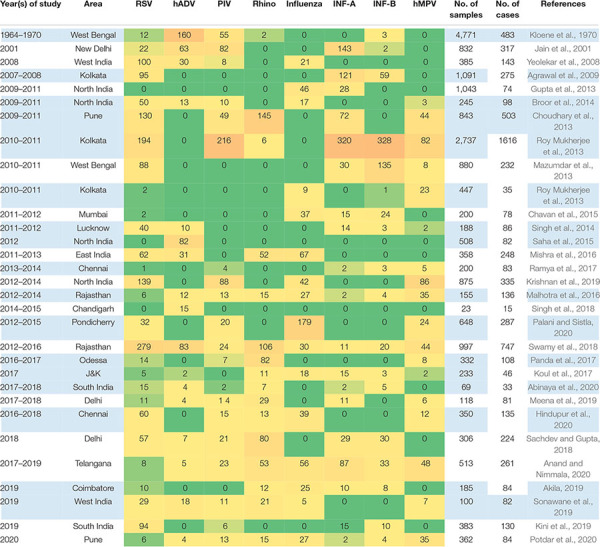

*The number of cases detected (Green to Orange Shading indicate increasing numbers) in the given study for a particular virus is listed under the virus name. RSV, respiratory syncytial virus; hADV, human adenovirus; PIV, parainfluenza virus; Rhino, rhinovirus; INF-A, influenza A; INF-B, influenza B; hMPV, human metapneumovirus.*

**FIGURE 1:**
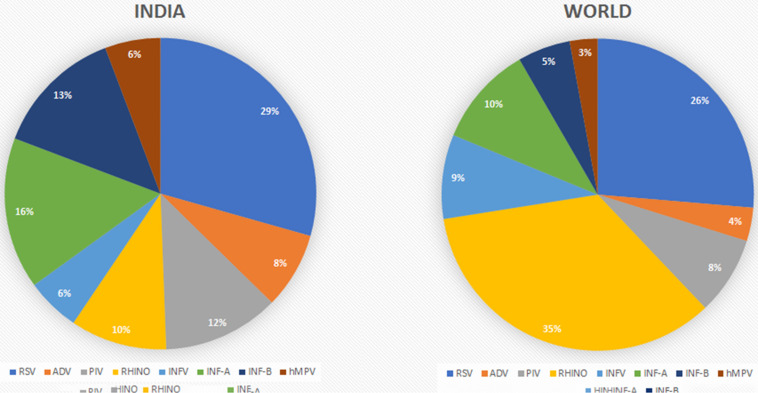
Comparison of the prevalence rates of viral infections between India and the rest of the world.

Table 1 and Figure 1 summarize the above findings with details of the type and number of viruses, their area of reporting, and the time durations from the overall findings of India. In total, RSV was found to be highly prevalent at all times with a percent contribution of 29%. Influenza A was the second most prevalent virus with a 16% share in all the detected viruses. The other viruses are also shown with their percent prevalence.

**FIGURE 2:**
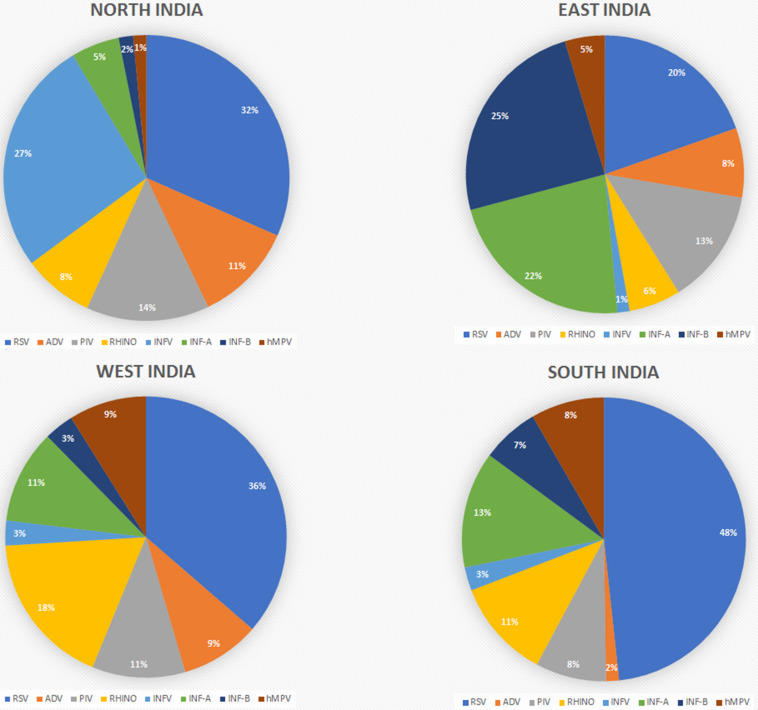
Prevalence of viral infections based on the geographical regions in India.

The reports were also classified based on the geographical regions as divided into four major zones, i.e., East, West, North, and South India. Table 2 and Figure 2 give an overview of the type and number of viruses and their prevalence based on the studied reports. The regions mentioned do not mean every part of the region, but that the area of the report falls under that geographical region. In brief, East India has reports from West Bengal and Odisha, and West India included reports from Maharashtra with independent reports from Mumbai and Pune along with Rajasthan. Reports from Ballabgarh, rural Ballabgarh, Chandigarh, Lucknow, J&K, Delhi, New Delhi, and Haryana were included in North India, while South India included reports from Karnataka with independent reports from Bengaluru, Puducherry, Telangana, and Tamil Nadu including Chennai and Coimbatore.

**TABLE 2:** Prevalence of virus infections based on the geographical regions in India (1970–2020).

Region	Virus type									
	
	RSV	ADV	PIV	RHINO	INFV	INF-A	INF-B	hMPV	Total no. of cases	No. of positive cases
North India	232	83	102	59	195	40	12	11	2,085	747
East India	468	193	320	144	34	532	584	111	1,2875	4,548
West India	469	118	138	230	37	139	44	115	2,448	1,351
South India	324	10	54	76	18	89	44	56	2,298	1,013
Total cases	1493	404	614	509	284	800	684	293	1,9706	7,659

*RSV, respiratory syncytial virus; ADV, adenovirus; PIV, parainfluenza virus; Rhino, rhinovirus; INFV, influenza virus; INF-A, influenza A; INF-B, influenza B; hMPV, human metapneumovirus.*

The reports were further classified based on the prevalence of respiratory viruses in those regions considering the differences in climatic conditions and the chance of finding a higher number of infections with a particular virus (Table 2 and Figure 2).

#### North India

The North India region is a large area with cold temperatures and chilling conditions in the winter. This could be the reason behind influenza and parainfluenza having detection rates of 27 and 14%, respectively. The 2,085 reported cases had 747 positive detections for upper respiratory tract infection (URTI; Jain et al., 2001; Broor et al., 2014; Singh et al., 2014; Saha et al., 2015; Sachdev and Gupta, 2018; Krishnan et al., 2019; Meena et al., 2019). Parainfluenza and INF-B cases were reported to be higher here.

#### East India

It was found that publications are mostly available for West Bengal, only Kolkata and Odessa. In total, these studies have examined 12,875 patients for respiratory tract infection, of which 4,548 were detected positive for viral infections. East India is the only region from which relatively lower reported cases of RSV (20%) were found. INF-A and INF-B show the highest prevalence rates in India, i.e., 22 and 25%, respectively (Kloene et al., 1970; Agrawal et al., 2009; Mazumdar et al., 2013; Roy Mukherjee et al., 2013; Mishra et al., 2016; Panda et al., 2017). Data analysis revealed that the number of reported cases and the prevalence of influenza are higher compared to those of other regions in India.

#### West India

**FIGURE 3:**
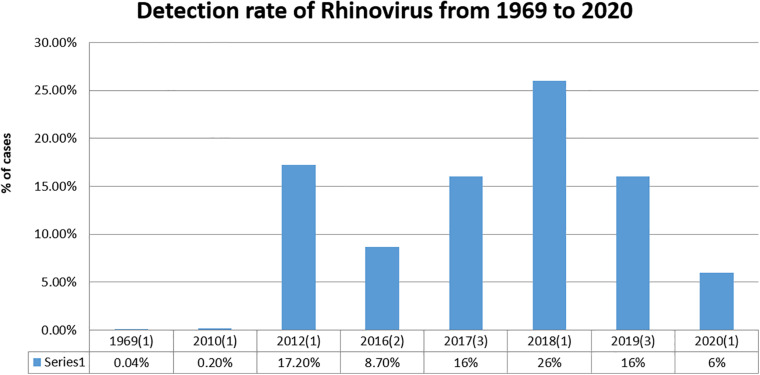
Detection rate of rhinovirus from 1964 to 2020. The number of reports included in the study during this period is indicated in brackets.

Reports from this region were mostly from Pune and Rajasthan, while other regions barely reported the viral diagnosis. As per these reports, 8,448 cases were studied and 1,315 samples were found positive for respiratory virus infections. Rhinovirus has a higher infection rate (18%) than in other regions and is detected regularly (Figure 3; Yeolekar et al., 2008; Choudhary et al., 2013; Chavan et al., 2015; Malhotra et al., 2016; Swamy et al., 2018; Sonawane et al., 2019). This region has reported the highest prevalence of rhinovirus: the cause of the common cold. RSV was also found to be higher in this region.

#### South India

The South India region with 2,298 cases reported 1,013 positive detections for respiratory virus, and around 50% of the cases detected were RSV, which is higher than that in any other region in the country (Akila, 2019; Kini et al., 2019; Abinaya et al., 2020; Anand and Nimmala, 2020; Hindupur et al., 2020; Palani and Sistla, 2020).

**FIGURE 4:**
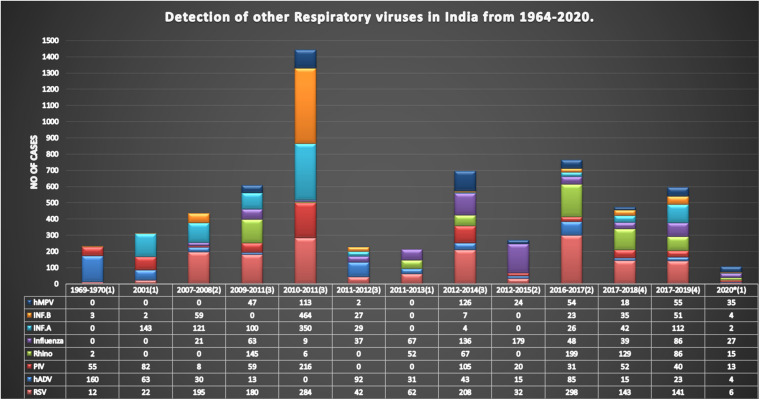
Prevalence of respiratory viruses from 1969 to 2020 in India. This figure shows the total prevalence of respiratory viruses in India from 1969 to 2020. The number of cases is reported on the *Y*-axis and the year of reporting is presented on the *X*-axis, while the number in brackets indicates the number of papers published in that period, with papers published in the same year being merged. The tabular format below the graphical presentation includes the number of viruses studied along with the year-wise reported positive cases.

The very first report that we considered has summarized data from four villages in West Bengal, and the sampling duration was from 1964 to 1966. More than 4,000 samples were collected and 625 viruses were isolated. These included all types of respiratory viruses, with parainfluenza, RSV, adenovirus, rhinovirus, and influenza virus types. It was discussed that there were waves of rising infections during this period, while the persistence of these viruses was also noted. The results of other studies, as described in Table 1 and Figure 4, can be discussed based on individual viruses, and their trend of detection in a given period is as described below.

#### Respiratory Syncytial Virus

From 1969 to 2001, reported cases of RSV were comparatively less than those of other viruses, but after 2007, the detection rate increased gradually, taking the total count of RSV-infected patients to 1,625 as calculated with the data included in this study. RSV has a higher prevalence rate in India than any other respiratory viruses, as shown in Figure 2. As compared regionally, South India showed a bit higher infection rate. This is the trend not only in India but also in other parts of the world (Figure 1). RSV has always been a part of the diagnostic machinery and was initially detected in moderate numbers routinely, except in 2009–2010 and 2012–2014 when its detection rate reached as high as 25% (Choudhary et al., 2013) and 41% (Krishnan et al., 2019), respectively, of the total positive cases. The prevalence took an upward trend afterward, and very high number of cases were reported until the recent time, except in 2020 when COVID-19 shadowed all detections and outnumbered all.

#### Human Adenovirus

Detection of adenovirus was available since the earlier period of the reporting in this study, and its prevalence was reported in higher numbers initially. In later publications, this was not given a high priority due to the non-severe outcomes of the infection and was not included routinely while diagnosing other respiratory viruses in many studies. After 2011, the availability of multiplex testing again allowed including adenovirus in the diagnostic panel; since then, it has been reported routinely, with moderate counts (Table 1 and Figure 4). Regional comparison showed its lower prevalence in South India, while the other three regions have a higher prevalence of this virus (Figure 2).

#### Human Parainfluenza Virus

Human parainfluenza viruses, a member of the Paramyxoviridae family, is classified as HPIV-1 to HPIV-4 based on the serotypes. In most cases of serious infections, HPIV-3 is diagnosed as a causal entity followed by HPIV-1 and HPIV-2, while HPIV-4 is associated with milder symptoms and is not included in the diagnosis for routine assays. The incidence rates of HPIV have always been higher whenever detected. The literature reported a higher incidence in colder temperatures; hence, more cases were recorded in North India and during the winter season compared to the other parts of the country and in other seasons (Table 1 and Figure 2).

#### Influenza Virus

In some of the papers, influenza virus was diagnosed and reported as influenza only and was not further classified into its subtypes; hence, it has been depicted as a separate row in Table 1, along with INF-A and INF-B. It has been reported to have moderate to high counts, except in 2011–2012 when there was an influenza outbreak and a higher number of samples were collected. As per the published literature, influenza was found to be one of the highly prevalent infections after 2001.

#### Influenza A

After RSV, influenza A shows a higher detection rate in India, and in the current review, a total of 929 confirmed cases are included (Figure 2). INF-A is highly detected in East and South India, maybe because of the pandemic. The counts of influenza viruses remain very high, as reported in various studies so far, despite the availability of the vaccine in 2015. However, there was a gradual decline observed after this period.

#### Influenza B

The detection rate of INF-B is relatively low as compared to other viruses, although a peak was detected in 2010–2011 due to the enhanced detection during the outbreak of H1N1. At that time, a total of 4,064 patients were tested under routine diagnosis, whereas 675 cases were detected positive for INF-B.

#### Human Metapneumovirus

In India, hMPV has been taken into consideration in routine diagnostic tests since 2009. Before that, no cases of hMPV have been detected, as depicted in Figure 4. The prevalence of hMPV in India remained low compared to the other viruses until 2012; thereafter, the number of positive cases rose slightly each year. The detection rate is higher in warmer regions, i.e., South and West India, compared to colder regions, i.e., North and East India (Figure 2).

### Rhinovirus: Is the Prevalence Increasing?

During our analysis, it was very apparent that rhinovirus has always been present in regional outbreaks or epidemics. Increased numbers of rhinovirus cases are being reported with improved diagnostics and better medical coverage. Although timelines could not be drawn with the limited data, we could observe a pattern that indicates increasing number of cases of rhinovirus infection in India, which is in line with global reports. Figure 3 shows the pattern of cases during the period from 1969 to 2020, leaving those years in which there were no reports published.

### The Scenario in Other Parts of the World

We also tried to compare our findings with reports from other parts of the world. We went through the literature available from other parts of the world and focused on studies reporting larger number of samples. Reports from five continents—Asia, Australia, Africa, Europe, and the United States—were found to be useful. At the same time, we included individual reports from the countries such as Bangladesh, China, Kenya, Thailand, Egypt, Italy, the Netherlands, Hong Kong, and Sweden, with 1971–2015 as the period of the research (Table 3). Overall, these case studies reported 36,559 cases. Cases from the different continents showed a diversity of regions, weather conditions, gender, race, age, religion, etc. (Henrickson et al., 2004; Farha and Thomson, 2005; Esposito et al., 2013; Zheng et al., 2018; Milucky et al., 2020). The reports that were picked up for this study gave cumulative data for a certain period and a larger coverage in terms of spread. Sporadic reports are not considered here as we aimed to get a generalized picture of the global scenario of all the respiratory viruses. WHO has conducted global surveillance of the important respiratory pathogens such as influenza and RSV. The Global Influenza Surveillance and Response System (GISRS) is one of the examples of the global surveillance of influenza, which has been done for more than 60 years and is still going on. Similarly, an RSV surveillance system is proposed using the GISRS platform. The National Respiratory and Enteric Virus Surveillance System (NREVSS) in the United States also conducts surveys of most of the respiratory viruses to understand the temporal and geographic circulation patterns by focusing on each virus individually. The patterns obtained from the published literature are shown in Table 4 and Figure 1. After analysis, it was observed that India and other regions of the world follow a similar trend of prevalence of viral infection. Rhinovirus is the most prevalent virus with 35% of cases worldwide, whereas in India rhinovirus has a detection rate of 11%, but the trend line shows that its prevalence is increasing (Figure 3). The prevalence of RSV was found to be very similar in India compared to other parts of the world. In India, RSV is the most prevalent virus, whereas it is the second most prevalent virus in other parts of the world. In India, influenza and its INF-A subtype show almost similar detection rates (influenza: 11% in India and 9% in other parts of the world; INF-A: 15% in India and 10% in other parts of the world). INF-B and hMPV have higher prevalence rates in India of 12 and 8%, respectively, whereas the worldwide incidence rate is only 3% for both INF-B and hMPV. The prevalence rates of ADV and PIV were found to be almost similar in India to other parts of the world. However, their detection rates are also low, as per the reviewed literature (Table 4).

**TABLE 3:** Representative studies from the rest of the world to compare the scenario of viral disease prevalence in India.

Area of reporting	hADV	RSV	Influenza	FLU.A	FLU.B	Rhino	PIV	hMPV	Year(s) of	Total no.	No. of
	hADV								research	of samples	positive cases
Wisconsin, United States	27	778	–	241	45	30	226	–	1996–1998	3,325	1,347
Finland, United States, Australia, Switzerland, the Netherlands, Hong Kong, Sweden	95	572	–	285	270	74	211	63	1986–2003	9,768	1,059
Europe, Asia, America, Oceania, Africa	126	561	246	–	–	2,330	150	90	1971–2014	20,486	3,503
Milan, Italy	11	188	57	6	8	144	11	49	2007–2014	592	435
Bangladesh, China, Guatemala, Kenya, Thailand, Egypt	27	72	417	327	127	272	70	36	2013–2015	2,388	1,815
Total no. of cases	286	2,171	720	859	450	2,850	668	238	1971–2015	36,559	8,159

*hADV, human adenovirus; RSV, respiratory syncytial virus; FLU.A, influenza A; FLU.B, influenza B; Rhino, rhinovirus; PIV, parainfluenza virus; hMPV, human metapneumovirus.*

**TABLE 4:** Comparison of the detection rates of viruses in India with those of other parts of the world.

Prevalence in India (%)	Virus	Prevalence in the rest of the world (%)
26	RSV	25
7	ADV	4
10	PIV	8
11	Rhino	35
11	Influenza	9
15	INF-A	10
12	INF-B	3
8	hMPV	3

*RSV, respiratory syncytial virus; ADV, human adenovirus; PIV, parainfluenza virus; Rhino, rhinovirus; INF-A, influenza A; INF-B, influenza B; hMPV, human metapneumovirus.*

## Discussion

Although previous attempts have been made to estimate the prevalence rates of respiratory viruses or specific viruses in some states of the country, there have been no report summarizing the burden of respiratory viruses or infectious diseases, deaths, across all states of India for a long time. In total, the scenario of the whole country can be discussed as depicted in Figure 2 and Table 1. RSV remained the most prevalent respiratory virus in India for the whole duration of the study period. If we add up all the influenza types together, we may find it to be the most prevalent viral cause of respiratory infections. Parainfluenza has also been a significant one, with rhinovirus as an emerging viral pathogen during this period, which may be due to improved diagnostics. At the same time, the prevalence rates of adenovirus and hMPV remained on the lower side.

RSV was the dominant one in all regions, except for East India that has variable percent contributions of all the other viruses, while influenza was highest in East India, followed by North India, compared to the other geographical regions. Reports from East India show higher numbers of influenza B, followed by influenza A, and a few reports indicating influenza as one group without details about its types. Also, this region has a higher prevalence of parainfluenza virus compared to all other regions. The higher prevalence rates of influenza and parainfluenza viruses in North and East India could be due to the extreme cold weather during the winter season and also to the drastic temperature variability in different seasons. Most of the studies considered all types of influenza in one group for reporting purposes; however, their subtypes may vary in occurrence and also may not be classified further due to resource constraints.

Human metapneumovirus had the lowest prevalence in all the regions, with the least prevalence in North India. West and South India have relatively higher prevalence rates of this virus. The higher percentages of hMPV in West and South India may be due to the relatively hot and humid climate in the majority of the areas, being in the coastal region. As reported earlier, infections with hMPV peak during the rainy season, and hence its higher prevalence in humid regions can be correlated (Evelyn et al., 2019). The western region has a relatively lower prevalence of influenza and other viruses compared to the other regions, while it has the highest number of reported cases of rhinovirus. The southern region was found to report the highest number of RSV cases, which also contributes to the overall high number of this virus in India.

The distribution of adenovirus was found to be uneven, with high prevalence in North India while very low in South India. A reason for this may be its moderate symptoms and low severity. Another possibility is that the infected individuals are normally not subjected to diagnosis, and hence many cases remain undiagnosed. In most cases, it was detected during surveys or after an attempt to detect other viral infections.

Rhinovirus has been the most prevalent virus in most parts of the world and is now also being reported frequently in India. The reason for the lower prevalence of rhinovirus in India may be its underdiagnosis in the earlier phase of infection (Figure 3). With improved and wide-ranging diagnostic procedures, it now seems that the pattern of its prevalence is similar to that of the rest of the world. The observation in this study also points toward an increase in prevalence from 2012 to 2019, after which COVID-19 has taken over and not many reports could be found. This also points toward the long persistence of this virus and a need to work in this area to avoid any major epidemic due to its variants or larger spread.

## Conclusion

The outcomes of this study have projected an excessive load of disability-adjusted life years (DALYs; 32% of the global count) due to respiratory infections compared to the total population of the country, which is 18% of the global population. We provided a broad evaluation of the burden of respiratory viruses and diseases in different states of India, as classified under four geographical regions from 1970 to 2020, based on all accessible data. An important finding of this study is that most states in India have higher rates of infections of RSV, influenza, and parainfluenza. These are the top three infectious viruses as per the studied reports. Also, it was found that from 2016 to 2017, infection of rhinovirus has increased at a higher rate. The exercise was done during the collection of data, and its compilation also indicated the unavailability of a systematic centralized database of respiratory viruses and their regional prevalence and cumulative yearly incidences. The inferences drawn out from the prevalence data can help in deciding the diagnostic priorities in these regions and devising local protocols for routine diagnosis. In conclusion, the review points toward the need for improved detection of multiple viruses and informed management of their infections. Another key finding during our data analysis was that most of the reports were originated from areas where research facilities are established. Hence, either the coverage of these research facilities may be expanded or a few more diagnostic research laboratories may be established in those areas that are remote and not accessed by the public so far for the diagnosis of these viruses. Also, routine surveillance of such viruses may be added to their mandate. A new ray of hope has been seen with the decision for the establishment of VRDLs in 2016 by the Department of Health Research (DHR)/Indian Council of Medical Research (ICMR). This is a network of laboratories to enhance the country’s capacity for the early diagnosis of all viral infections with public health importance in the Indian context. The target is to establish 160 such laboratories all over the country; 106 of such laboratories are already established and are either functioning or being made functional.

## Author Contributions

RW: manuscript draft preparation, review, and editing. SJ: review and approval. VN: concept, manuscript writing, editing, approval, and supervision. All authors contributed to the article and approved the submitted version.

## Conflict of Interest

The authors declare that the research was conducted in the absence of any commercial or financial relationships that could be construed as a potential conflict of interest.

## Publisher’s Note

All claims expressed in this article are solely those of the authors and do not necessarily represent those of their affiliated organizations, or those of the publisher, the editors and the reviewers. Any product that may be evaluated in this article, or claim that may be made by its manufacturer, is not guaranteed or endorsed by the publisher.

## Abbreviations

SARI: severe acute respiratory infection
RSV: respiratory syncytial virus
HRSV: human respiratory syncytial virus
HPIV: human parainfluenza virus
HRV: human rhinovirus
INF: influenza
HMPV: human metapneumovirus
HAdV: human adenovirus
DALY: disability-adjusted life year
WHO: World Health Origination
ALRI: acute lower respiratory infection
VRDLs: virus research and diagnostic laboratories.

## References

[B1] Abinaya S. Gaspar B. Benjamin A. (2020). A study on aetiology and outcomes of viral lower respiratory tract infections in hospitalized children from South India. *Sri Lanka J. Child Health* 49 218–222. 10.4038/sljch.v49i3.9137

[B2] Agrawal A. S. Sarkar M. Chakrabarti S. Rajendran K. Kaur H. Mishra A. C. (2009). Comparative evaluation of real-time PCR and conventional RT-PCR during a 2 year surveillance for influenza and respiratory syncytial virus among children with acute respiratory infections in Kolkata, India, reveals a distinct seasonality of infection. *J. Med. Microbiol.* 58 1616–1622. 10.1099/jmm.0.011304-0 19713363

[B3] Akila K. (2019). *Viral Etiology of Acute Respiratory Tract Infections in Adults in Tertiary Care Hospital*. Doctoral dissertation. Coimbatore: PSG Institute of Medical Sciences and Research.

[B4] Anand M. Nimmala P. (2020). Seasonal incidence of respiratory viral infections in Telangana, India: utility of a multiplex PCR assay to bridge the knowledge gap. *Trop. Med. Int. Health* 25 1503–1509. 10.1111/tmi.13501 32996228

[B5] Banerjee A. De P. Manna B. Chawla-Sarkar M. (2017). Molecular characterization of enteric adenovirus genotypes 40 and 41 identified in children with acute gastroenteritis in Kolkata, India during 2013-2014. *J. Med. Virol.* 89 606–614. 10.1002/jmv.24672 27584661

[B6] Briese T. Renwick N. Venter M. Jarman R. G. Ghosh D. Köndgen S. (2008). Global distribution of novel rhinovirus genotype. *Emerg. Infect. Dis.* 14 944–947. 10.3201/eid1406.080271 18507910 PMC2600308

[B7] Broor S. Dawood F. S. Pandey B. G. Saha S. Gupta V. Krishnan A. (2014). Rates of respiratory virus-associated hospitalization in children aged< 5 years in rural northern India. *J. Infect.* 68 281–289. 10.1016/j.jinf.2013.11.005 24269675 PMC7112698

[B8] Broor S. Parveen S. Maheshwari M. (2018). Respiratory syncytial virus infections in India: epidemiology and need for vaccine. *Indian J. Med. Microbiol.* 36 458–464. 10.4103/ijmm.ijmm_19_5 30880691

[B9] Chavan R. D. Kothari S. T. Zunjarrao K. Chowdhary A. S. (2015). Surveillance of acute respiratory infections in Mumbai during 2011-12. *Indian J. Med. Microbiol.* 33:43. 10.4103/0255-0857.148376 25560001

[B10] Choudhary M. L. Anand S. P. Heydari M. Rane G. Potdar V. A. Chadha M. S. (2013). Development of a multiplex one step RT-PCR that detects eighteen respiratory viruses in clinical specimens and comparison with real time RT-PCR. *J. Virol. Methods* 189 15–19. 10.1016/j.jviromet.2012.12.017 23313883 PMC7119668

[B11] Esposito S. Daleno C. Prunotto G. Scala A. Tagliabue C. Borzani I. (2013). Impact of viral infections in children with community-acquired pneumonia: results of a study of 17 respiratory viruses. *Influenza Other Respir. Viruses* 7 18–26. 10.1111/j.1750-2659.2012.00340.x 22329841 PMC5780730

[B12] Evelyn O. Jaime F. S. David M. Lorena A. Jenifer A. Oscar G. (2019). Prevalence, clinical outcomes and rainfall association of acute respiratory infection by human metapneumovirus in children in Bogotá, Colombia. *BMC Pediatr.* 19:345. 10.1186/s12887-019-1734-x 31601181 PMC6785857

[B13] Farha T. Thomson A. H. (2005). The burden of pneumonia in children in the developed world. *Paediatr. Respir. Rev.* 6 76–82. 10.1016/j.prrv.2005.03.001 15911451

[B14] Gupta V. Dawood F. S. Rai S. K. Broor S. Wigh R. Mishra A. C. (2013). Validity of clinical case definitions for influenza surveillance among hospitalized patients: results from a rural community in North India. *Influen. Other Respirat. Virus.* 7, 321–329. 10.1111/j.1750-2659.2012.00401.x PMC5779832 22804843

[B15] Henrickson K. J. Hoover S. Kehl K. S. Hua W. (2004). National disease burden of respiratory viruses detected in children by polymerase chain reaction. *Pediatr. Infect. Dis. J.* 23 S11–S18. 10.1097/01.inf.0000108188.37237.48 14730265

[B16] Hindupur A. Menon T. Dhandapani P. (2020). Molecular surveillance of respiratory viruses in children with acute respiratory infections in Chennai, South India. *Int. J. Infect. Dis.* 101:515. 10.1016/j.ijid.2020.09.1337

[B17] Jain N. Lodha R. Kabra S. (2001). Upper respiratory tract infections. *Indian J. Pediatr.* 68 1135–1138. 11838568 10.1007/BF02722930 PMC7091368

[B18] Kini S. Kalal B. S. Chandy S. Shamsundar R. Shet A. (2019). Prevalence of respiratory syncytial virus infection among children hospitalized with acute lower respiratory tract infections in Southern India. *World J. Clin. Pediatr.* 8 33–42. 10.5409/wjcp.v8.i2.33 31065544 PMC6477150

[B19] Kloene W. Bang F. B. Chakraborty S. M. Cooper M. R. Kulemann H. Ota M. (1970). A two-year respiratory virus survey in four villages in West Bengal, India. *Am. J. Epidemiol.* 92 307–320. 10.1093/oxfordjournals.aje.a121212 4319714

[B20] Koul P. A. Mir H. Akram S. Potdar V. Chadha M. S. (2017). Respiratory viruses in acute exacerbations of chronic obstructive pulmonary disease. *Lung India* 34 29–33. 28144057 10.4103/0970-2113.197099 PMC5234194

[B21] Krishnan A. Kumar R. Broor S. Gopal G. Saha S. Amarchand R. (2019). Epidemiology of viral acute lower respiratory infections in a community-based cohort of rural north Indian children. *J. Glob. Health* 9:010433. 10.7189/jogh.09.010433 PMC6513504 31131104

[B22] Malhotra B. Swamy M. A. Janardhan Reddy P. V. Gupta M. L. (2016). Viruses causing severe acute respiratory infections (SARI) in children = 5 years of age at a tertiary care hospital in Rajasthan, India. *Indian J. Med. Res.* 144 877–885. 10.4103/ijmr.IJMR_22_15 28474624 PMC5433280

[B23] Mazumdar J. Chawla-Sarkar M. Rajendran K. Ganguly A. Sarkar U. K. Ghosh S. (2013). Burden of respiratory tract infections among paediatric in and out-patient units during 2010-11. *Eur. Rev. Med. Pharmacol. Sci.* 17 802–808. 23609364

[B24] Meena J. P. Brijwal M. Seth R. Gupta A. K. Jethani J. Kapil A. (2019). Prevalence and clinical outcome of respiratory viral infections among children with cancer and febrile neutropenia. *Pediatr. Hematol. Oncol.* 36 330–343. 10.1080/08880018.2019.1631920 31512959

[B25] Milucky J. Pondo T. Gregory C. J. Iuliano D. Chaves S. S. McCracken J. (2020). The epidemiology and estimated etiology of pathogens detected from the upper respiratory tract of adults with severe acute respiratory infections in multiple countries, 2014–2015. *PLoS One* 15:e0240309. 10.1371/journal.pone.0240309 33075098 PMC7571682

[B26] Mishra P. Nayak L. Das R. R. Dwibedi B. Singh A. (2016). Viral agents causing acute respiratory infections in children under five: a study from Eastern India. *Int. J. Pediatr.* 2016:7235482. 10.1155/2016/7235482 PMC5149672 28018433

[B27] Narayan V. V. Iuliano A. D. Roguski K. Bhardwaj R. Chadha M. Saha S. (2020). Burden of influenza-associated respiratory and circulatory mortality in India, 2010-2013. *J. Glob. Health* 10:010402. 10.7189/jogh.10.010402 32373326 PMC7182391

[B28] Palani N. Sistla S. (2020). Epidemiology and phylogenetic analysis of respiratory viruses from 2012 to 2015–A sentinel surveillance report from union territory of Puducherry, India. *Clin. Epidemiol. Glob. Health* 8 1225–1235. 10.1016/j.cegh.2020.04.019 32346655 PMC7187823

[B29] Panda S. Mohakud N. K. Suar M. Kumar S. (2017). Etiology, seasonality, and clinical characteristics of respiratory viruses in children with respiratory tract infections in Eastern India (Bhubaneswar, Odisha). *J. Med. Virol.* 89 553–558. 10.1002/jmv.24661 27509268

[B30] Potdar V. Choudhary M. L. Bhardwaj S. Ghuge R. Sugunan A. P. Gurav Y. (2020). Respiratory virus detection among the overseas returnees during the early phase of COVID-19 pandemic in India. *Indian J. Med. Res.* 151 486–489. 10.4103/ijmr.ijmr_638_20 32474556 PMC7530434

[B31] Rafeek R. A. M. Divarathna M. V. M. Noordeen F. (2020). A review on disease burden and epidemiology of childhood parainfluenza virus infections in Asian countries. *Rev. Med. Virol.* 31:e2164. 10.1002/rmv.2164 32996257

[B32] Ramya R. Sagadevan P. JayaramJayaraj K. (2017). Genetic characterization and the development of multiplex PCR for common respiratory viruses, in Chennai during September 2013 to January 2014. *Int. J. Chemtech Res.* 10 389–401.

[B33] Rao B. L. Gandhe S. S. Pawar S. D. Arankalle V. A. Shah S. C. Kinikar A. A. (2004). First detection of human metapneumovirus in children with acute respiratory infection in India: a preliminary report. *J. Clin. Microbiol.* 42 5961–5962. 10.1128/jcm.42.12.5961-5962.2004 15583354 PMC535278

[B34] Roy Mukherjee T. Chanda S. Mullick S. De P. Dey-Sarkar M. Chawla-Sarkar M. (2013). Spectrum of respiratory viruses circulating in eastern India: prospective surveillance among patients with influenza-like illness during 2010–2011. *J. Med. Virol.* 85 1459–1465. 10.1002/jmv.23607 23765782 PMC7166942

[B35] Sachdev A. Gupta D. (2018). Incidence and severity profile of viral respiratory tract infections in children admitted to the tertiary level pediatric intensive care unit. *J. Pediatr. Crit. Care* 5:96. 10.21304/2018.0501.00315

[B36] Saha S. Pandey B. G. Choudekar A. Krishnan A. Gerber S. I. Rai S. K. (2015). Evaluation of case definitions for estimation of respiratory syncytial virus associated hospitalizations among children in a rural community of northern India. *J. Glob. Health* 5:010419. 10.7189/jogh.05.020419 PMC4652925 26649172

[B37] Salvi S. Kumar G. A. Dhaliwal R. S. Paulson K. Agrawal A. Koul P. A. (2018). The burden of chronic respiratory diseases and their heterogeneity across the states of India: the global burden of disease study 1990-2016. *Lancet Glob. Health* 6 E1363–E1374. 10.1016/S2214-109X(18)30409-1 30219316 PMC6227385

[B38] Singh A. K. Jain A. Jain B. Singh K. P. Dangi T. Mohan M. (2014). Viral aetiology of acute lower respiratory tract illness in hospitalised paediatric patients of a tertiary hospital: one year prospective study. *Indian J. Med. Microbiol.* 32:13–28. 10.4103/0255-0857.124288 24399381

[B39] Singh M. P. Ram J. Kumar A. Rungta T. Gupta A. Khurana J. (2018). Molecular epidemiology of circulating human adenovirus types in acute conjunctivitis cases in Chandigarh, North India. *Indian J. Med. Microbiol.* 36 113–115. 10.4103/ijmm.ijmm_17_258 29735838

[B40] Sonawane A. A. Shastri J. Bavdekar S. B. (2019). Respiratory pathogens in infants diagnosed with acute lower respiratory tract infection in a Tertiary Care hospital of Western India using multiplex real time PCR. *Indian J. Pediatr.* 86 433–438. 10.1007/s12098-018-2840-8 30637585 PMC7091426

[B41] Swamy M. A. Malhotra B. Reddy P. J. Tiwari J. (2018). Profile of respiratory pathogens causing acute respiratory infections in hospitalised children at Rajasthan a 4 year’s study. *Indian J. Med. Microbiol.* 36 163–171. 10.4103/ijmm.ijmm_18_84 30084405

[B42] van den Hoogen B. G. de Jong J. C. Groen J. Kuiken T. de Groot R. Fouchier R. A. (2001). A newly discovered human pneumovirus isolated from young children with respiratory tract disease. *Nat. Med.* 7 719–724. 11385510 10.1038/89098 PMC7095854

[B43] World Health Organization (2021). *World Health Organization fact sheet:Influenza (Seasonal).* Geneva: World Health Organization.

[B44] Worldometer (2021). Availble online at: https://www.worldometers.info/coronavirus/ (accessed July 24, 2021).

[B45] Yeolekar L. R. Damle R. G. Kamat A. N. Khude M. R. Simha V. Pandit A. N. (2008). Respiratory viruses in acute respiratory tract infections in Western India. *Indian J. Pediatr.* 75 341–345. 18536887 10.1007/s12098-008-0035-4

[B46] Zheng X. Y. Xu Y. J. Guan W. J. Lin L. F. (2018). Regional, age and respiratory-secretion-specific prevalence of respiratory viruses associated with asthma exacerbation: a literature review. *Arch. Virol.* 163 845–853. 10.1007/s00705-017-3700-y 29327237 PMC7087223

